# LIVING-APART-TOGETHER RELATIONSHIPS: EXPLORING OLDER ADULT BLACK WOMEN’S PERSPECTIVES ON CAREGIVING EXCHANGES

**DOI:** 10.1093/geroni/igz038.3325

**Published:** 2019-11-08

**Authors:** Nytasia Hicks

**Affiliations:** Scripps Gerontology Center, Miami University, Oxford, Ohio, United States

## Abstract

Recent research suggests that the preference for living-apart-together (LAT) relationships, where individuals are committed to one another but reside in separate households, has increased among older adults. However, older adult LAT couples prefer not to exchange care to maintain autonomy. In this study, we examine future expectations of spousal caregiving exchanges among older adult black women in LAT relationships. Eleven black women ages 60-74 (married and unmarried) completed two semi-structured phone interviews about future spousal caregiving expectations as to receiving and providing emotional, financial, and physical support. Regarding providing spousal care, findings from this qualitative descriptive study suggest that participants were consistent in their intent to provide emotional support, yet divided in visualizing themselves providing both financial and physical support for a spouse. As to receiving care from a spouse, participants envisioned their spouses providing emotional support, but responses were inconsistent on the expectation of spouses providing financial and physical support. Participants anticipate receiving emotional and physical support from their adult children and/or close family members instead of spouses. Autonomy, level of commitment, and the exchange of health information were also identified as core concepts. This study has implications for applications to different intimate relationships regarding living arrangements, to professionals supporting caregiving families, and to further studies of family gerontology.

